# Sign Language Recognition System for Deaf Patients: Protocol for a Systematic Review

**DOI:** 10.2196/55427

**Published:** 2025-01-23

**Authors:** Milena Soriano Marcolino, Lucca Fagundes Ramos Oliveira, Lucas Rocha Valle, Luiza Marinho Motta Santa Rosa, Zilma Silveira Nogueira Reis, Thiago Barbabela de Castro Soares, Elidéa Lúcia Almeida Bernardino, Raniere Alislan Almeida Cordeiro, Raquel Oliveira Prates, Mario Fernando Montenegro Campos

**Affiliations:** 1 Universidade Federal de Minas Gerais Medical School Belo Horizonte Brazil; 2 Telehealth Center University Hospital of Universidade Federal de Minas Gerais Belo Horizonte Brazil; 3 Institute for Health Technology Assessment (IATS) Porto Alegre Brazil; 4 Medical School Universidade Federal de Ouro Preto Ouro Preto Brazil; 5 Faculdade Ciências Médicas de Minas Gerais Belo Horizonte Brazil; 6 Faculty of Arts and Science Universidade Federal de Minas Gerais Belo Horizonte Brazil; 7 Department of Computer Science Universidade Federal de Minas Gerais Belo Horizonte Brazil

**Keywords:** computer neural networks, artificial intelligence, biomedical technology, communication aids for disabled, computer vision, sign language, hearing loss, deaf people, communication barriers, gestures

## Abstract

**Background:**

Individuals with hearing impairments may face hindrances in health care assistance, which may significantly impact the prognosis and the incidence of complications and iatrogenic events. Therefore, the development of automatic communication systems to assist the interaction between this population and health care workers is paramount.

**Objective:**

This study aims to systematically review the evidence on communication systems using human-computer interaction techniques developed for deaf people who communicate through sign language that are already in use or proposed for use in health care contexts and have been tested with human users or videos of human users.

**Methods:**

A systematic review will be performed based on a literature search in MEDLINE, Web of Science, ACM, and IEEE Xplore as well as top-tiered conferences in the area to identify relevant studies. The inclusion criteria are the description of the development of a sign language recognition system in a health care context and the testing with human users. Independent investigators (LFRO, LRV, and LMMSR) will screen eligible studies, and disagreements will be solved by a senior researcher (MSM). The included papers will undergo full-text screening. A PRISMA (Preferred Reporting Items for Systematic Reviews and Meta-Analysis) flow diagram will be presented to visually summarize the screening process, ensuring clarity and transparency in presenting the results. Additionally, a comprehensive chart table will be constructed to consolidate essential data related to the key variables extracted from the studies. These results will be meticulously analyzed and presented descriptively, offering insightful interpretations of the information encapsulated within the table.

**Results:**

A preliminary search was performed in April 2024. Researchers concluded the study selection by July 2024. Data extraction, synthesis, report, and recommendations are expected to be finished by February 2025.

**Conclusions:**

This systematic review will identify human-machine systems that enable communication in health services involving deaf patients, presenting the framework that includes usability and application in human contexts. We will present a comprehensive panel of findings, highlighting systems used to tackle communication barriers and offer a narrative comparison of current implementation practices.

**International Registered Report Identifier (IRRID):**

PRR1-10.2196/55427

## Introduction

The term “disabling hearing loss” refers to any type of hearing problem that is considered to be at least moderate (greater than 40 dB), including deafness, and that requires some type of health device to allow adequate communication with the individual [1]. According to the World Health Organization, approximately 430 million people, which accounts for over 5% of the world’s population, have some disabling hearing loss [2]. It is estimated that by 2050, over 700 million people will have some hearing impairment [2].

The scientific literature highlights numerous inequalities faced by the deaf community regarding access to health care services [3-5]. A qualitative study involving 93 deaf adults primarily communicating in American Sign Language revealed that most participants struggled with understanding medical instructions due to communication challenges [6]. Despite often being accompanied by family members during medical appointments, participants expressed dissatisfaction with their effectiveness as communication facilitators. They reported feeling excluded from conversations and expressed privacy concerns when using interpreters [6], a pertinent issue regarding the confidentiality of medical interactions.

In this context, it becomes evident that individuals with hearing impairment are more likely to experience preventable health issues, including adverse problems in acute care and chronic diseases, such as obesity, hypertension, cardiovascular disease, diabetes, smoking, and alcoholism [7,8]. A scoping review of 2022 revealed a gap in the interpretation of sign language for health care and showed that this area is underresearched [9]. Thus, several studies have been committed to the development of assistive technologies, using artificial intelligence techniques, aiming to mitigate the inequalities related to health accessibility experienced by the deaf community. Among these technologies, it is important to highlight that the creation of communication systems that allow translation between sign language and written text, has shown to be very promising [10]. However, despite all the progress attained, the current literature has also shown a huge gap regarding the use of these strategies with deaf people, especially in health care contexts. For this reason, it seems obvious that the creation and implementation of communication systems to enable better interaction between the deaf population and health care workers are paramount. This study aims to systematically review the evidence on communication systems using human-computer interaction techniques developed for deaf individuals who communicate through sign language that are already in use or proposed for use in health care contexts and have been tested with human users or videos of human users.

## Methods

### Study Design

This is a systematic review protocol. The study design is a systematic search of the medical and other technical literature followed by a narrative synthesis of the results. The research protocol was registered in the Open Science Framework (OSF) [11]. It followed guidance from the Cochrane Guidelines and the PRISMA (Preferred Reporting Items for Systematic Reviews and Meta-Analysis) statement [12]. The PRISMA-P (PRISMA Protocols) checklist can be seen in Multimedia Appendix 1.

A multidisciplinary team comprising researchers from health and computing domains, along with linguistic specialists in sign language, will collaboratively conduct the systematic review. Two members (ELAB and RAAC) of the team are linguistic specialists and sign language researchers, of whom one is deaf (RAAC). Both of them revised the terminology used in this review and revised the methodology critically to ensure that the needs of deaf people have been taken into account.

### Strategy for Preparing the Research

Phases of this review are as follows: (0) identification of the need for a review, (1) preparation of a proposal for a review, (2) development of a review protocol, (3) protocol registration in OSF, (4) identification of research, (5) selection of studies, (6) study quality assessment, (7) data extraction, (8) data synthesis, and (9) report and recommendations.

### Research Question

The primary research question is as follows: What technologies have been developed and tested in real-world settings to translate sign and oral languages, facilitating communication between deaf patients who primarily use sign language and health care workers?

The specific questions are as follows:

- Question 1: In which context of health care have these technologies been used?
- Question 2: Which languages (sign and oral) can these technologies translate?
- Question 3: Which technologies are required
  in loco
  for it to be used?
- Question 4: How were they developed?
- Question 5: How were they deployed and tested?
- Question 6: How has the communication between health care workers and deaf people been improved by using these technologies?
- Question 7: How was the efficacy of these technologies evaluated?
- Question 8: Is the system or technology “bidirectional”?

### Search Strategy

The search process will commence following registration on the OSF [10]. Independent reviewers will meticulously search the following databases: Web of Science, MEDLINE, IEEE Xplore, ACM, and Google Scholar.

A draft of the search strategy was developed by 5 of the authors (MSM, LFRO, LRV, ROP, and ZSNR), using Medical Subject Headings (MeSH), and text words related to the topic of interest were defined. The proposed search strategy is shown in Multimedia Appendix 2.

Studies published from the inception of the database onward will be inclusively considered, without imposing restrictions on the time frame or publication date, as well as language. Searches will be rerun before the final analysis to ensure comprehensiveness. Additionally, in the event of unpublished studies, authors will be contacted. Reference lists of eligible studies will be thoroughly explored to identify additional eligible studies.

Detailed search data for the identified studies and information for each phase will be presented in a flowchart, accessible in Multimedia Appendix 3, following the guidelines outlined in the PRISMA method [12].

### Eligibility Criteria

To be included in the systematic review protocol, a study must be prospective, retrospective, or descriptive; describe the development of communication systems applied to deaf people who communicate through sign language in a health care encounter context; and include testing with human users or videos of human users. The human users of the identified technologies should encompass individuals of any age who are deaf and primarily communicate through sign language.

Correspondences, short communications, and conference abstracts will also be included. Studies that do not align with the research questions specified earlier, fail to report use within health care encounters, do not involve testing with human users, or lack videos of human users will be excluded from the review.

### Outcome Definition

The primary anticipated outcome will consist of a compilation of reports detailing technologies using human-computer approaches aimed at facilitating communication for deaf patients during health care encounters. Secondary outcomes will involve conducting a narrative comparison of the identified technologies.

### Study Selection

In the screening phase, studies will be selected based on the predetermined inclusion and exclusion criteria after reviewing their titles, abstracts, and full texts. Researchers will independently screen titles and abstracts, with full-text review conducted if uncertainty arises from the abstract. Papers deemed potentially eligible based on title and abstract will undergo full-text screening for final eligibility determination. Eligible papers will be included in the study. Any disagreements regarding eligibility will be resolved by a senior member (MSM) of the team (MSM). The study selection process is represented using the PRISMA flow diagram, which can be found in Multimedia Appendix 3. Included studies will subsequently be selected for qualitative and quantitative analysis during the inclusion phase.

### Risk of Bias and Methodological Quality Analysis

The review will conduct a descriptive analysis. There is no specific risk of bias tool for the type of study we expect to find. If applicable, we use the mobile health evidence reporting and assessment checklist in the individual studies [13].

### Data Extraction and Data Synthesis

Independent reviewers (LFRO, LRV, and LMMSR) will extract data from the full text of eligible manuscripts using a predefined model. Any uncertainties or divergences will be resolved by a senior researcher (MSM). The following data items will be extracted from each publication: first author, journal and year of publication, country, the aim of the study, whether it was a multidisciplinary study team, corpus of terms used to develop the communication system, corpus examples, communication system development, intervention and testing, languages involved (oral and sign), evaluation or use in a real context, sample size (number of users), preconditions to use the technology and technology needed (hardware or software), development technology (artificial intelligence, imaging processing, etc), which health context (eg, general, emergency, and teleconsultation), technology readiness level (development and testing or if it is being marketed), testing: human users or video of human users, accuracy measures, metrics for evaluating communication effectiveness and parameters to assess to what extent innovation is addressing the communication needs of deaf persons, and ethical and software security issues. These data are detailed in Multimedia Appendix 4. Whenever possible, we will also seek to infer systems’ characteristics of flexibility and scalability in various health care settings. Additionally, the research team will email researchers from all individual studies included in the systematic review to update the current status of their systems and to ensure the accuracy and relevance of the information presented in the systematic review. A qualitative analysis of the usability results will be performed for the review.

Results will be presented in a PRISMA flow diagram and a descriptive format (using tables and diagrams, if necessary) aligned with the objective and scope of the review. Results will be presented to carry out a descriptive analysis of the information in the table. For each variable extracted, a narrative summary will be provided, describing how the results relate to the review’s objective.

### Data Management

The abstracts and full text of manuscripts identified from the search will be uploaded to Rayyan software from Qatar Computing the Research Institute, a software developed specifically to expedite the screening of titles, abstracts, and manuscripts using a process of semiautomation [14]. After uploading the search results, duplicates will be removed in the Rayyan environment [15]. A data extraction form will be developed in Microsoft Excel and piloted before use.

### Description of Statistical Methods and Software Used

If feasible, we intend to conduct statistical analysis to aggregate accuracy measures. However, we anticipate that this approach may not be sufficient. In such instances, the systematic review will proceed with a narrative review of the evidence, providing a qualitative synthesis of the findings.

### Ethical Considerations

The systematic review does not require ethics approval, as all data used will be provided from published documents. The authors are committed to reporting any outcomes, even the unexpected ones, to ensure transparency and responsibility.

## Results

A preliminary search was performed in April 2024. Researchers concluded the study selection by July 2024. Data extraction, synthesis, report, and recommendations are expected to be finished by February 2025. The review timeline is shown in Multimedia Appendix 5.

## Discussion

### Expected Findings

Communication barriers persist for deaf individuals in health care, urging solutions. In this context, this review aims to summarize the current literature on communication systems using human-computer interaction techniques for sign language users, in a health care context. As the main finding, we aim to reveal the current state of communication systems dedicated to improving health care interactions with deaf people and using a variety of advanced models to enhance accuracy and usability. Additionally, we seek to identify existing gaps in the development of these systems. We anticipate that the predominant focus on technical aspects or innovations may lead to a scarcity of manuscripts addressing communication outcomes, which should ideally be the primary focus, as it determines the effectiveness of communication.

### Strengths

Sign language recognition is often considered less advanced compared to other recognition systems like facial or speech recognition. Several characteristics are important to enhance communication with deaf people using systems and devices, such as adequate screens, stable internet connection, qualified and trained professionals, interoperability of the system, safety for the patient and professional, and extensive testing [9]. Research in this field must focus on models that improve the accuracy of recognition systems and, more importantly, communication. To investigate these factors, a multidisciplinary staff including health, linguistic, and technology teams will participate in the search process, conducting and validating its progress. This would strengthen the results, as it broadens the results landscape. Another important strength to highlight is that the systems assessed in this review will not depend on a translator, as is the case for video remote interpretation. We believe that, if proven effective, these systems could enhance the autonomy of deaf individuals in managing their health care.

Furthermore, these systems have the potential to surmount barriers related to patient privacy concerns and communication challenges. With the escalating integration of artificial intelligence techniques in health care, there is growing recognition that capturing signs or gestures and converting them into audio or written language, and vice versa, can significantly enhance accessibility to health care services, ranging from primary care facilities to emergency departments. Identifying information regarding the availability, usability, and implementation specifics of such systems stands as a crucial outcome to be unearthed.

In response to this urgent demand, our research group is diligently conducting a comprehensive and high-caliber systematic review. While the ultimate goal must consider context and innovation, it is paramount to prioritize meeting the needs of the ultimate end users: individuals who are deaf.

To identify and delineate these systems, along with associated contextual possibilities, use methods, required equipment, and technological development and testing, we will present a comprehensive findings panel and conduct a narrative comparison. This approach aims to facilitate a comprehensive overview and deeper understanding of the current state of the art. Our results are poised to offer valuable insights into the landscape of systems and the evolution of human-computer–assistive technologies used in aiding deaf patients.

In emergency care units, timely access to information is crucial for defining patient routes, conducting necessary examinations, and providing critical interventions and advice. Previous studies have shown that preventable adverse events are more likely to occur in cases with communication problems, particularly in emergency situations [7]. Despite the pressing need for studies in this area, testing interventions in emergency contexts is more complex, leading us to anticipate fewer analyses in this critical domain.

### Limitations and Future Directions

This systematic review, while intended to be comprehensive, may encounter limitations impacting its conclusions. First, the diversity of sign language dialects, and the specificity of their use, might limit the generalizability of the findings across different cultural contexts. Additionally, the rapid evolution of technology in the field of sign language recognition systems may also mean that some of the reviewed technologies quickly become outdated.

With the results of this review, we expect to advocate future directions for the creation of more adaptive and inclusive communication systems, capable of learning from a broader spectrum of sign language dialects and styles. However, it specifically targets individuals who use sign language. This approach may not represent the entire deaf community, as some individuals may prefer lip reading, writing, or other forms of communication due to unfamiliarity with or reluctance to use sign language. Further studies should explore technologies aimed at enhancing communication for individuals with varying degrees of hearing impairment and differing abilities in sign language proficiency. Expanding research in this direction can contribute significantly to addressing the diverse communication needs within the deaf and hard-of-hearing community.

Our team plans to disseminate the protocol and findings of this research in forums with public authorities, academies, and the population to present solutions for improving effective communication in health services, especially involving the most vulnerable populations. In addition, as social media has a relevant impact on our society, all the results will be posted on social media as well to be available to the population.

## Appendix

Multimedia Appendix 1 PRISMA-P (Preferred Reporting Items for Systematic Review and Meta-Analysis Protocols) checklist.

Multimedia Appendix 2 Search strategy.

Multimedia Appendix 3 PRISMA (Preferred Reporting Items for Systematic Reviews and Meta-Analysis) flowchart.

Multimedia Appendix 4 The variables analyzed in the systematic review.

Multimedia Appendix 5 Gantt chart for the project timetable.

## Abbreviations

OSF: Open Science Framework
MeSH: Medical Subject Headings
PRISMA: Preferred Reporting Items for Systematic Reviews and Meta-Analysis
PRISMA-P: Preferred Reporting Items for Systematic Reviews and Meta-Analysis Protocols

## Data Availability

All data generated or analyzed during this study are included in this published paper and its supplementary information files.
