# Supplementary material for: Sign Language Recognition System for Deaf Patients: Protocol for a Systematic Review
Source: JMIR Res Protoc. 2025 Jan 23;14:e55427. doi: 10.2196/55427 (PMC11803331; doi:10.2196/55427)
Supplement: Multimedia Appendix 2 [file resprot_v14i1e55427_app2.docx]

Medline:

("Neural Networks, Computer"[Mesh] OR "Artificial Intelligence"[Mesh] OR "Biomedical Technology"[Mesh] OR "Communication Aids for Disabled"[Mesh] OR "Image Processing, Computer Assisted"[Mesh] OR "Machine Learning"[Mesh] OR "assistive technolog*") AND ("Sign Language"[Mesh] OR "Hearing Loss"[Mesh] OR "Persons With Hearing Impairments"[Mesh] OR "Communication Barriers"[Mesh] OR "Gestures"[Mesh])

IEEE, ACM:

(“Assistive technology” OR “Artificial Intelligence” OR “Computational Intelligence” OR “Machine Intelligence” OR “Computer Reasoning” OR “Computer Vision System” OR “Knowledge Acquisition” OR “Machine Intelligence” OR “Machine Learning” OR “Deep Learning” OR “Computer Neural Network” OR “Neural Network Model” OR “Perceptron” OR “Connectionist Model” OR “Neural Network” OR “Communication Aid for Disabled” OR “Speech Synthesizer” OR “Biomedical Technology” OR “Health Technology” OR “Health Care Technology” OR “Image Processing” OR “Computer-Assisted Image Analysis” OR “Computer Assisted Image Analysis”) AND (“Sign Language” OR “Hearing Loss” OR “Deafness” OR “Deaf”)

Web of science:

(“Assistive technolog*” OR “Artificial Intelligence” OR “Computational Intelligence” OR “Machine Intelligence” OR “Computer Reasoning” OR “Computer Vision System*” OR “Knowledge Acquisition” OR “Machine Intelligence” OR “Machine Learning” OR “Deep Learning” OR “Computer Neural Network*” OR “Neural Network Model*” OR “Perceptron*” OR “Connectionist Model*” OR “Neural Network*” OR “Communication Aid* for Disabled” OR “Speech Synthesizer” OR “Biomedical Technolog*” OR “Health Technolog*” OR “Health Care Technolog*” OR “Image Processing” OR “Computer-Assisted Image Analys*” OR “Computer Assisted Image Analys*”) AND (“Sign Language” OR “Hearing Loss” OR “Deafness” OR “Deaf”)
