# Supplementary material for: Sign Language Recognition System for Deaf Patients: Protocol for a Systematic Review
Source: JMIR Res Protoc. 2025 Jan 23;14:e55427. doi: 10.2196/55427 (PMC11803331; doi:10.2196/55427)
Supplement: Multimedia Appendix 4 [file resprot_v14i1e55427_app4.docx]

**Variables Analyzed:**

We will extract the following data items from each publication:

Study Characteristics:

- First author.
- Bibliographic item and year of publication.
- Country in which the study was conducted.
- Aim of study.

Study Team:

- Whether it was a multidisciplinary team. If so, from which fields were the professionals that comprised the team?
- Is there a specific framework for collaboration among the diverse fields?

Software Characteristics:

- Corpus used to develop the communication system.
- Corpus examples.
- Communication system development, intervention and testing.
- Languages and dialects involved (oral and sign).
- Whether it captures facial expressions.
- Whether the communication can be bidirectional.
- Whether the software development took into account any particular need related to children or the elderly.
- Training needed for efficient usage.
- How dependable is the system.
- How up-to-date is the system.
- Data safety and privacy issues (if videos of users are deleted after translation; if videos of users are employed to improve the system upon authorization, and if so, what measures are taken to preserve patient confidentiality and to avoid privacy breaches).
- Safety standards to ensure quality translation and interoperability of oral and signed languages (e.g. does it take oral/sign language differences into account when translating?)

Ethical Issues:

- Whether the study provides information about how laws and rules affect the accessibility and caliber of the communication system, as well as system usability.

We will extract data on how usability was assessed in the following categories:

- Was the evaluation/use performed in a real context?
- Whether and how these technologies were adopted and used over time.
- Sample size (number of users).
- Participants’ recruitment criteria (online vs. offline).
- Description of the technical proficiency of the users.
- Development technology (artificial intelligence, computer vision, etc).
- Which health context (e.g. general, emergency, teleconsultation, etc).
- Whether the system manages emergencies.
- Pre-conditions for the use of the technology (infrastructure required) and Internet/Computer literacy.
- Application access (download; payment).
- Technology requirements (hardware and software necessary for deployment).
- Technology Readiness Level (what is the technological maturity of the solution?).
- Testing: human users/video of human users; laboratory or real-world environment.
- Accuracy measures.
- Metrics of usage (logins, log file analysis).
- Translation time (in each direction, if the system translates in both directions).
- The ease of use (e.g. does it need previous training? What guidelines should healthcare staff follow while receiving training in connection with these communication systems?).
- Reliability (if the system crashes; bug fixes; recovery time).
- Instructions and recommendations given to the user.
- Whether and how qualitative feedback was obtained for user experience (strengths and shortcomings of the application) - patient and health professional.
- Statistics of system usage (logins, log file analysis).
- Input for system enhancement (whether the researchers asked deaf users and professionals for points of improvement, and how frequently this feedback was given).
- Increased independence in health care management (e.g. reducing the need for intermediaries, such as translators).

Metrics for evaluating communication effectiveness, reported both by the deaf and the healthcare professional:

- What methodology was used to evaluate the system.
- Which criteria were used to evaluate communication improvement (with the system versus without the system).
- Whether the metrics were objective or subjective.
- Communication effectiveness (whether the communication was adequate as assessed both by the deaf and the healthcare professional).
- Which problems in communication were reported.

Additional information that will be extracted:

- Psychological effects of using the software.
