# Supplementary material for: Sign Language Recognition System for Deaf Patients: Protocol for a Systematic Review
Source: JMIR Res Protoc. 2025 Jan 23;14:e55427. doi: 10.2196/55427 (PMC11803331; doi:10.2196/55427)
Supplement: Multimedia Appendix 5 [file resprot_v14i1e55427_app5.docx]

|  | **2023** | | | | | | | | |
| --- | --- | --- | --- | --- | --- | --- | --- | --- | --- |
|  | **Apr** | **May** | **Jun** | **Jul** | **Aug** | **Sep** | **Oct** | **Nov** | **Dec** |
| **Preparation** |  |  |  |  |  |  |  |  |  |
| **Development of review protocol** |  |  |  |  |  |  |  |  |  |
| **Identification of research** |  |  |  |  |  |  |  |  |  |
| **Selection of studies** |  |  |  |  |  |  |  |  |  |

|  | **2024** | | | | | | | | | | | |
| --- | --- | --- | --- | --- | --- | --- | --- | --- | --- | --- | --- | --- |
|  | **Jan** | **Feb** | **Mar** | **Apr** | **May** | **Jun** | **Jul** | **Aug** | **Sep** | **Oct** | **Nov** | **Dec** |
| **Development**  **of review**  **protocol** |  | ***** | ***** |  |  |  |  |  |  |  |  |  |
| **Identification**  **of research** |  |  |  |  |  |  |  |  |  |  |  |  |
| **Selection of**  **studies** |  |  |  |  |  |  |  |  |  |  |  |  |
| **Data extraction**  **and synthesis** |  |  |  |  |  |  |  |  |  |  |  |  |
| **Report and**  **recommendations** |  |  |  |  |  |  |  |  |  |  |  |  |

* This period refers to amendments in the review protocol as suggested by JMIR Research Protocol reviewers. These amendments were included in the registration in the Open Science Framework.
